# Peak oxygen uptake is a strong prognostic predictor for pulmonary hypertension due to left heart disease

**DOI:** 10.1186/s12872-022-02574-0

**Published:** 2022-03-31

**Authors:** Xiu-Jun Zhong, Rong Jiang, Lu Yang, Ping Yuan, Su-Gang Gong, Qin-Hua Zhao, Ci-Jun Luo, Hong-Ling Qiu, Hui-Ting Li, Rui Zhang, Jing He, Lan Wang, Jie Tang, Jin-Ming Liu

**Affiliations:** 1 Department of Cardio-Pulmonary Circulation, Shanghai Pulmonary Hospital, Tongji University School of Medicine, No. 507 Zhengmin Road, Yangpu District, Shanghai, 200433 China; 2 Department of Respiratory Medicine, Yueyang Hospital of Integrated Traditional Chinese and Western Medicine, Shanghai University of Traditional Chinese Medicine, Shanghai, 200437 China; 3 Department of Cardiology, Second Affiliated Hospital of Nanchang University, 1 Minde Road, Nanchang, 330006 China

**Keywords:** Pulmonary hypertension due to left heart disease, Prognosis, Peak oxygen consumption, Combined post- and precapillary pulmonary hypertension, Isolated postcapillary pulmonary hypertension

## Abstract

**Background:**

Pulmonary hypertension in left heart disease (PH-LHD), which includes combined post- and precapillary PH (Cpc-PH) and isolated postcapillary PH (Ipc-PH), differs significantly in prognosis. We aimed to assess whether cardiopulmonary exercise testing (CPET) predicts the long-term survival of patients with PH-LHD.

**Methods:**

A single-center observational cohort enrolled 89 patients with PH-LHD who had undergone right heart catherization and CPET (mean pulmonary arterial pressure > 20 mm Hg and pulmonary artery wedge pressure ≥ 15 mm Hg) between 2013 and 2021. A receiver operating characteristic curve was plotted to determine the cutoff value of all-cause death. Survival was estimated using the Kaplan–Meier method and analyzed using the log-rank test. The Cox proportional hazards model was performed to determine the association between CPET and all-cause death.

**Results:**

Seventeen patients died within a mean of 2.2 ± 1.3 years. Compared with survivors, nonsurvivors displayed a significantly worse 6-min walk distance, workload, exercise time and peak oxygen consumption (VO_2_)/kg with a trend of a lower oxygen uptake efficiency slope (OUES) adjusted by Bonferroni’s correction. Multivariate Cox regression revealed that the peak VO_2_/kg was significantly associated with all-cause death after adjusting for Cpc-PH/Ipc-PH. Compared with Cpc-PH patients with a peak VO_2_/kg ≥ 10.7 ml kg^−1^ min^−1^, Ipc-PH patients with a peak VO_2_/kg < 10.7 ml kg^−1^ min^−1^ had a worse survival (*P* < 0.001).

**Conclusions:**

The peak VO_2_/kg is independently associated with all-cause death in patients with PH-LHD. The peak VO_2_/kg can also be analyzed together with Cpc-PH/Ipc-PH to better indicate the prognosis of patients with PH-LHD.

**Supplementary Information:**

The online version contains supplementary material available at 10.1186/s12872-022-02574-0.

## Introduction

Pulmonary hypertension (PH) due to left heart disease (LHD) is a major problem in patients with heart failure (HF) and the most common type of PH [1, 2]. The presence of PH suggests a poor prognosis and exercise capacity in patients with HF [1, 3] and LHD [4]. Recent studies have shown no treatment benefit in this population[2, 5]. PH-LHD is divided into combined post- and precapillary PH (Cpc-PH) and isolated postcapillary PH (Ipc-PH). Cpc-PH indicates the presence of precapillary components, which are associated with increased mortality [1]. The two subgroups are usually distinguished by several hemodynamic variables detected by a right heart catheterization (RHC). These variables include the transpulmonary gradient (TPG), pulmonary vascular resistance (PVR), and diastolic pressure gradient (DPG). Based on current guidelines in this field, PH-LHD defined as the mean pulmonary artery pressure (mPAP) > 20 mmHg and pulmonary artery wedge pressure (PAWP) > 15 mmHg at rest. Ipc-PH and Cpc-PH can be distinguished based on the PVR, Cpc-PH is characterized by an increased PVR of ≥ 3 WU [5].

Considering its invasiveness and the possibility of data distortion, RHC alone will likely be insufficient to assess PH-LHD patients [2]. In addition to RHC, other noninvasive techniques may be required in patients with PH-LHD. Modern CPET systems allow the analysis of gas exchange throughout exercise. An important practical significance of CPET is that it provides data concerning outcome prediction [6], which has usually been used to predict the severity and progression of HF [7]. The peak oxygen consumption (VO_2_) is the most well-established variable of CPET and has been considered a significant predictor of death in patients with HF [8]. A comprehensive analysis of the peak VO_2_, carbon dioxide output (VCO_2_), and ventilation (VE) is helpful to accurately predict the mortality of HF patients [9, 10].

PH-LHD is related to decreased exercise tolerance, and the degree of exercise impairment is directly correlated with disease severity [11]. However, CPET has not been widely used in clinical practice with PH-LHD, primarily due to poor knowledge of its potential and evidence. In the present study, we aimed to investigate whether the modified diagnostic criteria of hemodynamics for Ipc-PH and Cpc-PH were related to clinical outcomes, to study the incremental prognostic information provided by CPET, to estimate the prognostic value of these indices and to identify reliable prognostic factors for PH-LHD.

We present the following article in accordance with the STROBE reporting checklist.

## Methods

### Study design and patient population

We reviewed incident patients with suspected PH associated with LHD referred to our center between July 2013 and May 2020. Finally, 89 patients underwent CPET and RHC for hemodynamic evaluation were included. And all patients were followed up to January 31, 2021. The clinical characteristics and hemodynamic and CPET data were obtained during routine clinical care and were collected from hospital records. Demographic variables such as sex, age, body mass index, World Health Organization functional class (WHO FC), N-terminal pro-B type natriuretic peptide (NT-proBNP) and 6-min walk distance (6MWD) were obtained at baseline.

The patient inclusion criteria were as follows: (1) a diagnosis of LHD confirmed by experienced specialists according to the appropriate guidelines [5], including heart failure with a preserved left ventricular ejection fraction (LVEF) (HFpEF), heart failure with a reduced LVEF (HFrEF), valvular heart disease (VHD) and congenital/acquired cardiovascular conditions leading to postcapillary PH [5]; (2) After adequate medical treatment such as cardiotonic diuresis. RHC and CPET were performed (within one week) when patients were stable at not-acute decompensation period; and 3) PH-LHD defined as mPAP > 20 mmHg and PAWP > 15 mmHg at rest [7, 12]. Furthermore, PH-LHD was classified as Cpc-PH and Ipc-PH defined by PVR ≥ 3 Wood units (WU) and PVR < 3 WU, respectively [7, 12].

Patients were excluded for the following reasons: (1) a diagnosis of other PH groups as per the NICE criteria [13]; (2) no valid baseline CPET; (3) acute decompensated heart failure, severe cardiogenic shock requiring inotropic support or urgent mechanical circulatory support; (4) a lack of CPET or RHC; and (5) comorbidities such as severe chronic lung diseases and pulmonary embolism.

The study was conducted in accordance with the Declaration of Helsinki (as revised in 2013). The study was approved by the Institutional Ethics Committee of Shanghai Pulmonary Hospital approved the protocol (K16-317) and individual consent for this retrospective analysis was waived.

## Procedures

### Right heart catherization

RHC was performed as described previously using the Swan-Ganz catheter (7- or 7.5-Fr; Edwards Lifesciences LLC, Irvine, CA) [14]. The baseline hemodynamic variables evaluated included mPAP, right atrial pressure (RAP), PAWP, cardiac output (CO) and PVR. DPG = diastolic PAP—mean PAWP and TPG = mPAP—mean PAWP.

### Cardiopulmonary exercise testing

CPET was performed using an electromagnetically braked cycle ergometer (Master Screen CPET, Jaeger Crop., Hoechberg, Germany), and gas exchange data were recorded over 10-s intervals via a breath-by-breath system. The protocol consisted of 3 min of rest, followed by 3 min of unloaded pedalling at 60 revolutions per minute, subsequently, a progressively increasing workload of 10–25 W/min to the maximum tolerance and finally 5 min of recovery. A test was terminated if any of the following conditions were observed: fatigue, dyspnea, chest tightness, or any other uncomfortable feeling reported by the patient. Measurements included the exercise time, workload, O_2_ consumption, oxygen pulse (O_2_ pulse), end-tidal partial pressure of CO_2_ (P_ET_ CO_2_), minute ventilation, carbon dioxide output, VE/VCO_2_, VO_2_/VE, oxygen uptake efficiency plateau (OUEP), and the oxygen uptake efficiency slope (OUES).

The VO_2_, P_ET_ CO_2_, VE/VCO_2_, VO_2_/VE, and O_2_ pulse values at peak exercise were measured according to the highest 30-s averaged value obtained during peak exercise. The lowest VE/VCO_2_ was calculated by averaging the 9 lowest consecutive 10-s-averaged data points of VE/VCO_2_. The VE/VCO_2_ slope was obtained from linear regression analysis of the relationship of VE with VCO_2_. The oxygen uptake efficiency plateau was at 90 s for the highest consecutive values of VO_2_ (ml/min)/VE (L/min) [15]. Using linear square regression, we computed the oxygen uptake efficiency slope according to the following equation: VO_2_ = a × lgVE + b (‘a’ is OUES) [15].

### Outcome assessment

The primary outcome was all-cause death. All the patients were followed up until death or through January 31, 2021, whichever occurred first. Patients lost during follow-up were censored as alive on the last day of contact. We had an established PH database at our center. The data were obtained during follow-up or by telephone interview, and specific events were confirmed through medical records, death certificates or confirmation provided by immediate family members.

### Statistical analysis

All the results were expressed as means ± SD or medians (and interquartile range) for continuous variables and as the absolute number for categorical variables. Comparisons in the two groups (survivors and nonsurvivors) were performed using independent-samples *t*-test and the Mann–Whitney U test for parametric and nonparametric data, respectively. Differences in categorical variables between groups were assessed using χ^2^ test. Comparisons in the four groups were performed using ANOVA and the Kruskal–Wallis test for parametric and nonparametric data, respectively.

The Cox proportional hazards model was performed to determine the associations between the clinical indices and survival with or without covariate adjustment. A receiver operating characteristic (ROC) curve was used to select the cutoff value for independent predictors with the maximum sensitivity and specificity. Correlations were assessed by Spearman’s correlation coefficient. The Kaplan–Meier method and log-rank test were used to perform survival analyses. The Bonferroni method for correcting the significance level for multiple comparisons was applied. For all analyses, statistical significance was indicated by a 2-sided *P* < 0.05. The data were analyzed using SPSS 19.0 (SPSS Inc., Chicago, IL, USA).

## Results

### Characteristics and hemodynamic parameters between nonsurvivors and survivors

**Fig. 1:**
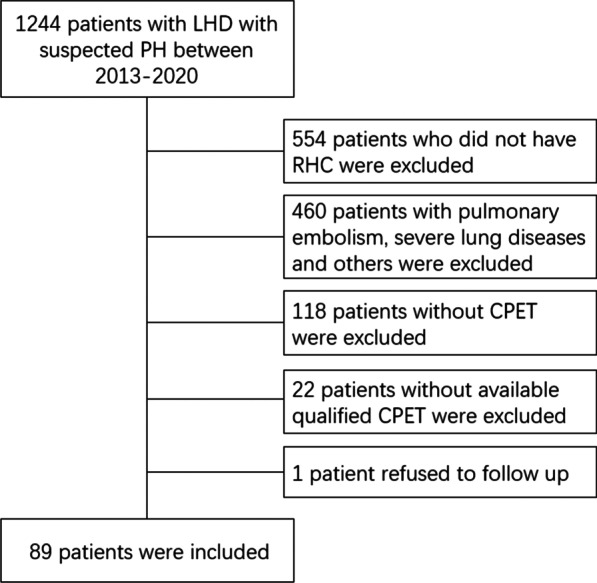
Flow chart of screening patients

A total of 89 eligible patients were included in this study, including 46 patients with Cpc-PH and 43 patients with Ipc-PH. The screening protocol is shown in Fig. 1. During a median follow-up of 3.0 (1.4, 4.2) years, all-cause mortality occurred in 17 (19.1%) patients. The follow-up rate was 95.5%. The patients had an average age of 64.0 (56.0, 72.5) years, and 35 patients (39.3%) were male. The demographics, baseline characteristics and hemodynamics were compared between survivors and nonsurvivors among the PH-LHD patients (Table 1). Significant differences were found between survivors and nonsurvivors regarding WHO-FC, 6MWD, NT-proBNP, renal function, PVR and DPG. However, when Bonferroni's correction of the significance level (*P* < 0.05) was applied, the adjusted significance level was 0.002. Compared with survivors, nonsurvivors walked a significantly shorter distance (*P* = 0.001).

**Table 1:** Comparison of the demographic characteristics and hemodynamic parameters between survivors and nonsurvivors

	Nonsurvivors (n = 17)	Survivors (n = 72)	*P*-value*
Age, years	69 (62.5,74.0)	63 (53.3, 72.0)	0.061
Male, n (%)	6 (35.3)	29 (40.3)	0.705
BMI, kg/m^2^	26.02 (21.5,27.1)	23.12 (21.1, 27.4)	0.266
WHO-FC, n (%)			0.045
I–II	1 (5.8)	21 (29.2)	
III-IV	16 (94.2)	51 (70.8)	
6 MWD, m	280 (187.5, 366.5)	410 (325.0, 453.8)	0.001
NT-pro-BNP, pg/ml	1802 (1032.0, 2736.5)	856 (387.5, 1908.5)	0.006
HFrEF, n (%)	1 (5.8)	3 (4.2)	0.759
HFpEF, n (%)	13 (76.5)	39 (54.2)	0.093
VHD^#^, n (%)	3 (17.6)	30 (41.7)	0.065
*Comorbidities, n (%)*			
Emphysema	8 (47.1)	26 (36.1)	0.403
AF	4 (23.5)	23 (31.9)	0.497
Hypertension	7 (41.2)	30 (41.7)	0.971
Diabetes	3 (17.6)	12 (16.7)	0.923
Renal insufficiency	5 (29.4)	5 (6.9)	0.008
*Medication*			
Diuretics	17 (100)	68 (94.4)	0.320
Anti-arrhythmias	4 (23.5)	19 (26.4)	0.809
Anti-hypertensive	7 (41.2)	30 (41.7)	0.971
*Echocardiography*			
RATD, cm	4.3 (3.8,5.6)	4.2 (3.8,5.0)	0.381
RVEDTD, cm	3.7 (3.1,4.2)	3.6 (3.0,3.9)	0.440
TAPSE, mm	1.7 (1.6,2.2)	1.8 (1.6,2.1)	0.532
*Pulmonary hemodynamics*			
sPAP, mmHg	64.0 (48.5,92.5)	55.0 (45.0, 67.8)	0.141
dPAP, mmHg	22.0 (15.5,30.0)	18.0 (15.0, 24.0)	0.134
mPAP, mmHg	42.0 (28.0,48.0)	34.0 (28.0, 42.0)	0.139
PAWP, mmHg	18.0 (15.5,20.5)	18.0 (16.0, 22.0)	0.543
PVR, Wood U	4.8 (2.5,6.4)	2.9 (2.0, 4.1)	0.023
DPG, mmHg	2.0 (− 5.0,10.5)	− 1.0 (− 3.0, 3.0)	0.029
TPG, mmHg	17.0 (12.0,32.5)	14.5 (10.0, 20.0)	0.086
CO, L/min	4.7 (4.1,5.6)	5.2 (4.1, 6.0)	0.334

The data are shown as the mean ± SD, n (%) or median (quartile range). BMI, body mass index; WHO-FC, World Health Organization function class; 6MWD, 6-min walk distance; HFrEF, heart failure with reduced ejection fraction; HFpEF, heart failure with preserved ejection fraction; VHD, valvular heart disease; AF, atrial fibrillation; RATD, right atrial transverse dimension; RVEDTD, right ventricular end-diastolic transverse dimension; TAPSE, tricuspid annular plane systolic excursion; sPAP, systolic pulmonary artery pressure; dPAP, diastolic pulmonary artery pressure; mPAP, mean pulmonary artery pressure; PAWP, pulmonary artery wedge pressure; PVR, pulmonary vascular resistance; DPG, diastolic pulmonary pressure gradient; TPG, transpulmonary gradient; CO, cardiac output. * When the Bonferroni method was employed to correct the significance level for 22 comparisons made in this study, the adjusted significant level was 0.002. ^#^VHD included moderate or severe mitral or aortic stenosis or insufficiency

### Comparison of CPET between nonsurvivors and survivors

A significant difference was observed in the workload, peak O_2_ pulse, exercise time, peak VO_2_, lowest VE/VCO_2_, peak VE/VCO_2_, OUEP and OUES between nonsurvivors and survivors (Table 2). Regarding exercise capacity, nonsurvivors had a worse workload, exercise time and peak VO_2_ (the adjusted significance level was 0.004) after applying Bonferroni's correction of the significance level (*P* < 0.05). In terms of ventilatory and gas exchange efficiency, a trend was observed toward a lower OUES in nonsurvivors (*P* = 0.009).

**Table 2:** Comparison of the CPET parameters between survivors and nonsurvivors

	Nonsurvivors (n = 17)	Survivors (n = 72)	*P*-value*
*Exercise capacity*			
Workload, watts	34.4 ± 17.0	59.2 ± 33.5	0.004
Peak O_2_ pulse, ml/beat	5.3 (3.5, 6.7)	6.2 (5.1, 8.0)	0.041
Exercise time, s	170.0 (110.0,220.0)	249.0 (181.5,290.0)	0.001
Peak VO_2_, mL/min/kg	9.4 ± 2.2	12.9 ± 3.3	< 0.001
*Ventilatory and gas exchange efficiency*			
Lowest VE/VCO_2_	43.2 (36.0, 46.0)	37.4 (33.1, 43.4)	0.026
VE/VCO_2_ slope	36.4 (31.4, 51.3)	32.5 (28.8, 38.0)	0.113
Peak VE/VCO_2_	46.5 (38.1, 49.2)	38.1 (34.5, 45.7)	0.022
Peak P_ET_ CO_2_, mmHg	31.3 ± 6.4	33.8 ± 6.7	0.179
Peak VO_2_/VE, mL/L	22.7 ± 5.3	25.4 ± 5.7	0.082
OUEP, mL/L	25.8 ± 4.9	28.5 ± 4.9	0.042
OUES	1.0 ± 0.3	1.3 ± 0.5	0.009

The data are shown as the mean ± SD or median (quartile range). CPET, cardiopulmonary exercise test; Cpc-PH, post- and precapillary pulmonary hypertension; Ipc-PH, isolated postcapillary pulmonary hypertension; VO_2_, oxygen consumption; VE/VCO_2_, minute ventilation/carbon dioxide output; P_ET_ CO_2_, end-tidal partial pressure of CO_2_; VO_2_/VE, oxygen uptake/minute ventilation; OUEP, oxygen uptake efficiency plateau; OUES, oxygen uptake efficiency slope. * When the Bonferroni method was employed for correcting for the significance level for 11 comparisons made in this study, the adjusted significant level was 0.004

### Factors influencing survival

**Fig. 2:**
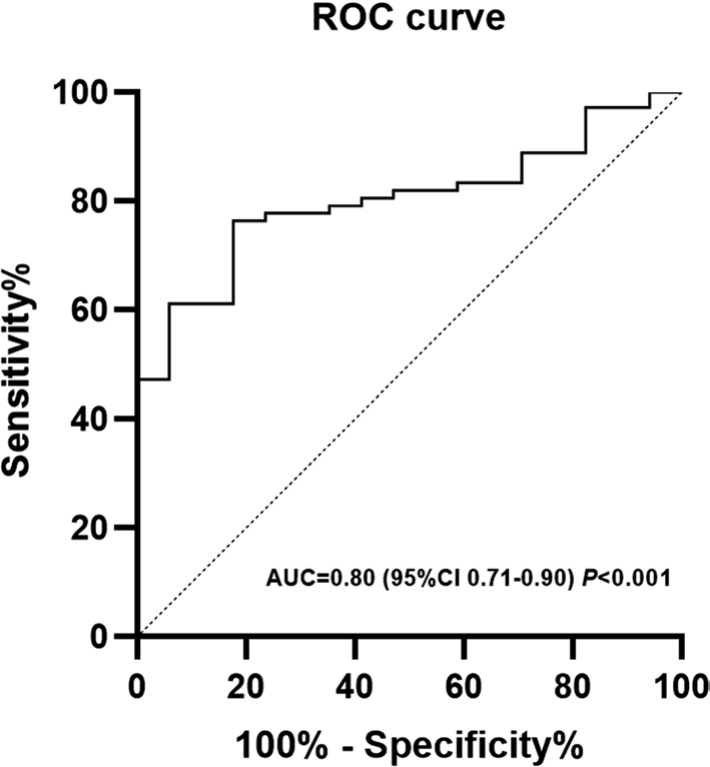
ROC curves to demonstrate the sensitivity and specificity of the peak VO_2_/kg for death in PH-LHD. ROC, receiver operating characteristic; AUC, area under the ROC curve; VO_2_, oxygen uptake; PH-LHD, pulmonary hypertension due to left heart disease

In the univariate Cox proportional hazards analysis (Table 3), age, 6MWD, Cpc-PH or Ipc-PH, exercise time, peak VO_2_/kg, lowest VE/VCO_2_, and OUES were significant predictors of death. Subsequently, all factors with a *P* value < 0.05 were included in the multivariate forward stepwise analysis, revealing that the peak VO_2_/kg was a significant independent predictor of all-cause death (hazard ratio: 0.487; 95% CI: 0.354–0.653; *P* < 0.001) after adjusting for Cpc-PH or Ipc-PH. The peak VO_2_/kg ≥ 10.7 ml kg^−1^·min^−1^ exhibited 76.4% sensitivity and 82.4% specificity with an area under the ROC curve of 0.8 (95% CI: 0.71 to 0.9; *P* < 0.001) (Fig. 2).

**Table 3:** Cox regression analysis for all-cause death in patients with PH-LHD

	Univariate analysis				Multivariate-Adjusted Analysis*			
Variables	HR	95% CI		*P*-value	HR	95% CI		*P*-value
		Lower	Higher			Lower	Higher	
Age, years	1.052	1.001	1.105	0.044				
6 MWD, m	0.996	0.993	0.999	0.008				
NT-proBNP**, pg/mL	2.341	1.407	3.894	0.001				
Cpc-PH/Ipc-PH	0.350	0.123	0.997	0.049				
Exercise Time, s	0.991	0.987	0.996	< 0.001				
Peak VO_2_/kg, mL/min/kg	0.532	0.411	0.689	< 0.001	0.487	0.359	0.660	< 0.001

PH-LHD, pulmonary hypertension due to left heart disease; 6MWD, 6-min walk distance; Cpc-PH, post- and precapillary pulmonary hypertension; Ipc-PH, isolated postcapillary pulmonary hypertension; VO_2_, oxygen consumption. * According to the rule of statistical power and Bonferroni correct, 6WMD, exercise time and Peak VO_2_/kg were finally reserved in the multivariate-adjusted analysis. ** NT-proBNP was log transformed

### Correlation between CO, 6MWD, NT-proBNP and peak VO_2_/kg

**Fig. 3:**
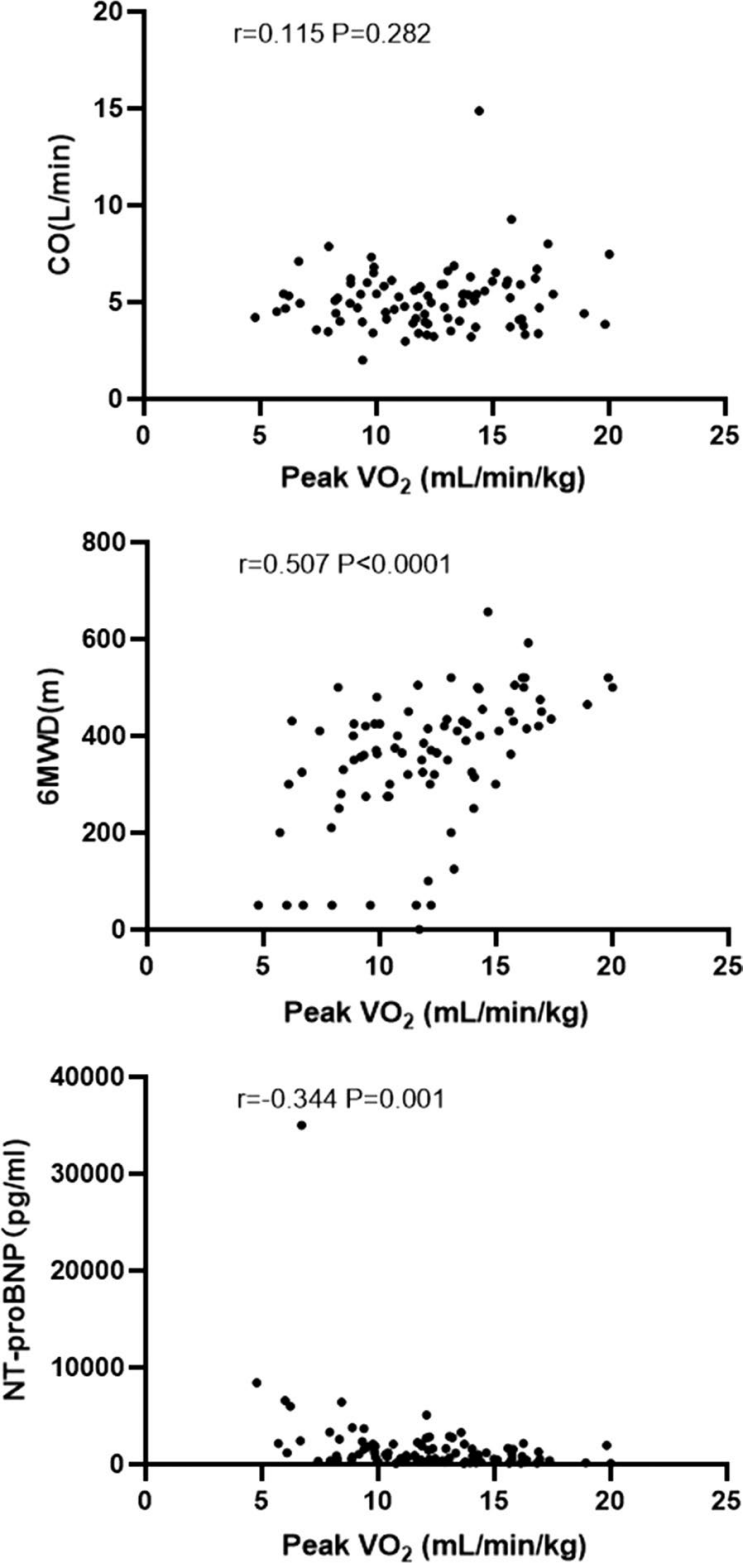
Correlation between CO, 6MWD, NT-proBNP and peak VO_2_/kg. VO_2_, oxygen uptake; CO, cardiac output; 6MWD, 6-min walk distance; NT-proBNP, N-terminal pro-B type natriuretic peptide

As shown in Fig. 3, there was no significant correlation between peak VO_2_/kg and CO (r = 0.115, *P* = 0.282), peak VO_2_/kg was positively correlated with 6MWD (r = 0.507, *P* < 0.0001), and peak VO_2_/kg was negatively correlated with NT-proBNP (r =  − 0.344, *P* = 0.001).

### Kaplan–Meier survival analysis

**Fig. 4:**
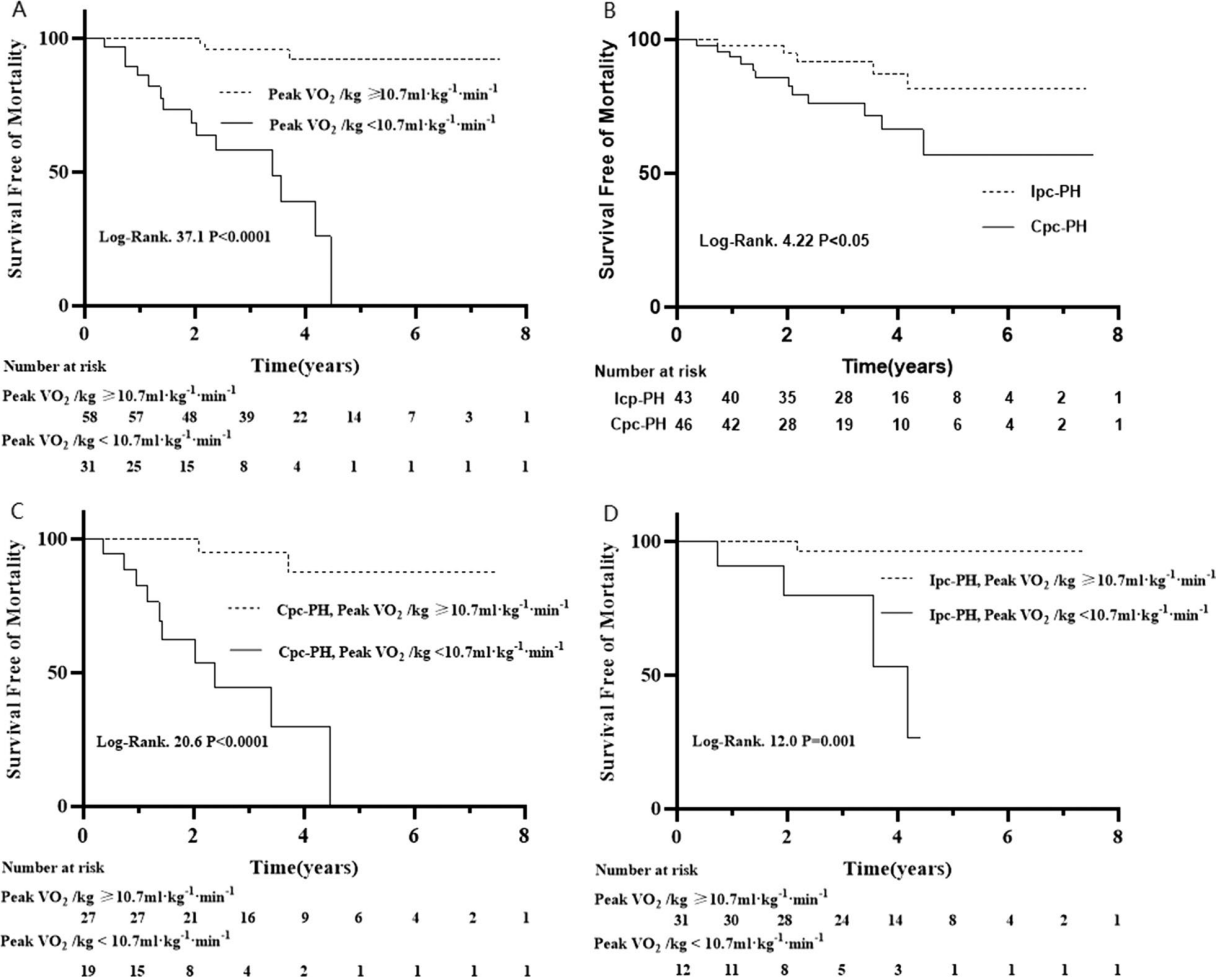
Kaplan–Meier analysis in PH-LHD, Cpc-PH and Ipc-PH patients stratified by peak VO_2_/kg. **A** Survival in PH-LHD patients between the peak VO_2_/kg ≥ 10.7 ml kg^−1^ min^−1^ and peak VO_2_/kg < 10.7 ml kg^−1^ min^−1^. **B** Survival in PH-LHD patients between Cpc-PH and Ipc-PH. **C** Survival in Cpc-PH patients between the peak VO_2_/kg ≥ 10.7 ml kg^−1^ min^−1^ and peak VO_2_/kg < 10.7 ml kg^−1^ min^−1^. **D** Survival in Ipc-PH patients between the peak VO_2_/kg ≥ 10.7 ml kg^−1^ min^−1^ and peak VO_2_/kg < 10.7 ml kg^−1^ min^−1^. Survival analyses were compared by the log-rank test. VO_2_, oxygen uptake; PH-LHD, pulmonary hypertension due to left heart disease; Cpc-PH, post- and precapillary pulmonary hypertension; Ipc-PH, isolated postcapillary pulmonary hypertension

Patients with a peak VO_2_/kg ≥ 10.7 ml kg^−1^·min^−1^ had a much better prognosis than those with a peak VO_2_/kg < 10.7 ml·kg^−1^ min^−1^ in PH-LHD patients (*P* < 0.0001) (Fig. 4A). Compared with Ipc-PH patients, Cpc-PH patients showed a worse survival (*P* < 0.05) (Fig. 4B). The prognosis of patients with a peak VO_2_/kg ≥ 10.7 ml kg^−1^ min^−1^ was better than that of those with a peak VO_2_/kg < 10.7 ml·kg^−1^ min^−1^ in Cpc-PH (*P* < 0.0001) (Fig. 4C). The prognosis of patients with a peak VO_2_/kg ≥ 10.7 ml kg^−1^ min^−1^ was better than that of patients with a peak VO_2_/kg < 10.7 ml·kg^−1^ min^−1^ in Ipc-PH (*P* = 0.001) (Fig. 4D). Additionally, hemodynamics and CPET parameters were significantly different among the above groups (Additional file 1: Table S1).

## Discussion

To our knowledge, there are few studies to explore the prosgnostic values of CPET for the mortality of patients with PH-LHD. For patients with PH-LHD, PVR is more significant to explain the prognosis based on peak VO_2_/kg. Our study demonstrated that the peak VO_2_/kg was independently associated with all-cause death in patients with PH-LHD. The peak VO_2_/kg can also be analyzed together with Cpc-PH/Ipc-PH, which can better indicate the prognosis of patients with PH-LHD. Nonsurvivors with PH-LHD had a worse 6MWD, workload, exercise and peak VO_2_/kg than survivors, revealing that PH-LHD patients with obvious exercise limitation had a poorer prognosis.

Although the current definition and classification of PH-LHD are based on hemodynamics, the application of hemodynamic parameters in prognostication is limited [16]. In addition to hemodynamic indices, other nonhemodynamic markers, including CPET profiles, can better determine the prognosis of patients with PH-LHD [5]. Further clinical studies are encouraged to better understand prognostic predictors. To the best of our knowledge, this study is the first to compare the invasive parameters of PH-LHD and CPET to study the predictors of mortality since the new standard was formulated in 2018 [16]. Significant differences were found in the CPET and hemodynamic parameters among the four groups according to the peak VO_2_/kg and Cpc-PH/Ipc-PH. Both the peak VO_2_/kg and Cpc-PH/Ipc-PH affected the prognosis, but the peak VO_2_/kg was better. The combination of the two could better predict the prognosis of patients with PH-LHD.

The presence of precapillary components in PH-LHD, defined as Cpc-PH, may consistently influence the prognosis [17]. However, using PVR alone to identify Cpc-PH, indicating the presence of precapillary components, remains controversial [5, 18]. Our previous study showed that DPG does not provide additional CPET information for patients with Cpc-PH beyond that provided by PVR [19], supporting those patients with Cpc-PH and Ipc-PH were differentiated according to PVR in the prognosis study of CPET. There have been conflicting results in the search for ideal prognostic indicators for patients with PH-LHD. PVR was considered to significantly, mildly or not predict the outcome in patients with PH-LHD [12, 16, 20, 21]. Our results showed that Cpc-PH with PVR ≥ 3 WU had a slight predictive effect on prognosis.

RHC plays an important role in distinguishing hemodynamic subtypes in patients with LHD, namely, Cpc-PH and Ipc-PH, but it is often inferior to CPET in accurately evaluating the functional status and prognostic information [22]. The data obtained from CPET have a recognized key role in the prognosis of HF [23], whether alone [24] or combined with non-CPET parameters [9, 25]. The application of an optimal CPET response in the risk stratification of mortality or other outcomes in patients with HF is controversial [26]. The peak VO_2_ describes the existence of functional impairment, its absolute value is used to grade the severity of exercise limitation in cardiac disease patients [22], and it is a well-established prognostic indicator in patients with HF. Some studies have shown that the VE/VCO_2_ relationship is a stronger predictor of mortality than the peak VO_2_ [9, 27–29]. In this study, we demonstrated that ventilatory and gas exchange CPET parameters predict survival in patients with PH-LHD. The more prognostic parameter is the VE/VCO_2_ rather than other parameters of ventilatory impairment. Different from the study of Mayer et al. [30], lowest VE/VCO_2_ was more meaningful than VE/VCO_2_ slope, but the VE/VCO_2_ related parameters were not as good as peak VO_2_. Among all CPET parameters, the peak VO_2_ was the best parameter to predict the death of patients with PH-LHD. This finding was similar to that reported in the HF population [31]. To our knowledge, few studies have explored the prognostic significance of the peak VO_2_ in invasively characterized PH-LHD. The peak VO_2_ is a broader marker of the severity and prognosis of heart and lung diseases.

Although peak VO_2_ has been studied for HF, no study has evaluated the impact of peak VO_2_ on the prognosis of Cpc-PH and Ipc-PH. In our study, there was no significant correlation between peak VO_2_ and cardiac output, but it was correlated with 6MWD and NT-proBNP, which indirectly supported that the decrease of peak VO_2_ in PH-LHD reflected more a general condition than simple hemodynamic disorder. Exercise capacity, whether assessed during CPET or walking tests (peak VO_2_ or 6MWD, respectively), is a recognized predictor of survival in HF and PAH [32]. The 6MWD contains important prognostic information [22, 33], similar to our results. Some studies have also shown that the 6MWD had only weak and nonsignificant prognostic power [34]. Groepenhoff et al*.*[32] found that the prognostic information of the 6MWD was better than that of the peak VO_2_ in PH patients, contrasting our results.

CPET parameters have become a new prognostic tool for PAH patients. Additionally, CPET provides a comprehensive pathophysiological assessment of patients with exercise restriction and dyspnea and is recommended for all patients with clinically stable PH [22]. In PAH patients, the peak VO_2_ and PVR are powerful independent prognostic indicators, and their combination can obtain the best risk stratification [34]. These different methods may be complementary in the risk stratification of PAH patients. Similarities and differences are observed among different types of PH. Our results also showed that the combination of the peak VO_2_ and Cpc-PH/Ipc-PH could better distinguish the significance of CPET and hemodynamic parameters and predict the prognosis. The peak VO_2_ is an independent and strong predictor of survival in PH-LHD patients. Cpc-PH/Ipc-PH, although also an accurate predictor, provides no independent prognostic information. This finding is similar to previous study findings on primary pulmonary hypertension, although the hemodynamic parameters are different [35]. Regardless of Ipc-PH or Cpc-PH, all PH-LHD patients with a peak VO_2_ < 10.7 ml·kg^−1^·min^−1^ at baseline had a higher risk of death. The peak VO_2_/kg < 10.7 ml·kg^−1^·min^−1^ is stronger than PVR ≥ 3WU in predicting prognosis, likely increasing the controversy of PVR alone. We suspect that mortality of PH-LHD is not only determined by hemodynamic factors caused by pulmonary hypertension, but also by the basic physical condition of patients. Peak VO_2_ is only an overall indicator of this pathophysiological state. Therefore, it is expected that the peak VO_2_ in PH-LHD is stronger than PVR in predicting the prognosis of PH-LHD.

Our study confirms that nonsurvivors of PH-LHD show a significantly decreased exercise capacity. Cpc-PH patients have a worse outcome than Ipc-PH patients. In our patient population, the prognostic value of the peak VO_2_ was better than that of the Cpc-PH/Ipc-PH, 6MWD and other CPET parameters. Our study suggests that hemodynamic variables need to be combined with assessment of cardiopulmonary exercise capacity when trying to determine individual risk in patients with PH-LHD.

Our study has some limitations. First, the prognostic effects of the peak VO_2_ and other CPET parameters were evaluated only once during the trial run. We did not evaluate any possible treatment changes during the follow-up or considered the impact of repeated CPET on the prognosis. Second, this study was performed at a single-center with a limited sample size, which may have provided less relevant evidence than a large sample and multicenter clinical research. Third, the retrospective design had selection bias, and this could have possibly led to a bias. The results of our study could have been influenced by the following selection bias. First of all, among the patients we excluded who did not undergo RHC, some refused invasive examination for fear or because echocardiography results were nearly normal after treatment. Others were too ill or old for invasive examination. For these patients with worse cardiopulmonary ability, our results may be overestimated. Secondly, among patients with other diseases excluded, such as severe lung diseases, these complications worsen patients' cardiopulmonary capacity, so our results may be overestimated. Finally, among the excluded patients without CPET or qualified CPET, they had the same standardized diagnosis and treatment procedure as the included patients. They may have similar age and sex distributions, with little possibility of selection bias. Prospective investigations of a large number of patients in the future will allow extensive and powerful multivariate analysis. Finally, we enrolled few patients with HFrEF in the present study, possibly leading to a survival bias.

## Conclusions

The peak VO_2_/kg is independently associated with all-cause death in patients with PH-LHD. The peak VO_2_/kg can also be analyzed together with Cpc-PH/Ipc-PH to better indicate the prognosis of patients with PH-LHD.

## Supplementary Information

**Additional file 1:** Comparison of CPET and hemodynamics stratified by sex and Cpc-PH or Ipc-PH.

## Data Availability

The data underlying this article will be shared on reasonable request to the corresponding author.

## Abbreviations

PH: Pulmonary hypertension
LHD: Left heart disease (−)
Cpc-PH: Combined post- and precapillary PH
Ipc-PH: Isolated postcapillary PH
CPET: Cardiopulmonary exercise testing
VO_2_: Oxygen consumption
OUES: Oxygen uptake efficiency slope
HF: Heart failure
RHC: Right heart catheterization
TPG: Transpulmonary gradient
PVR: Pulmonary vascular resistance
DPG: Diastolic pressure gradient
mPAP: Mean pulmonary artery pressure
PAWP: Pulmonary artery wedge pressure
VCO_2_: Carbon dioxide output
VE: Ventilation
WHO FC: World Health Organization functional class
NT-proBNP: N-terminal pro-B type natriuretic peptide
6MWD: 6-Minute walk distance
LVEF: Left ventricular ejection fraction
HFpEF: Preserved LVEF
HFrEF: Reduced LVEF
VHD: Valvular heart disease
WU: Wood units
RAP: Right atrial pressure
CO: Cardiac output
P_ET_ CO_2_: End-tidal partial pressure of CO_2_
OUEP: Oxygen uptake efficiency plateau
ROC: Receiver operating characteristic
