# Supplementary material for: Peak oxygen uptake is a strong prognostic predictor for pulmonary hypertension due to left heart disease
Source: BMC Cardiovasc Disord. 2022 Mar 31;22:137. doi: 10.1186/s12872-022-02574-0 (PMC8974096; doi:10.1186/s12872-022-02574-0)
Supplement: Supplementary file 1 — Additional file 1: Comparison of CPET and hemodynamics stratified by sex and Cpc-PH or Ipc-PH. [file 12872_2022_2574_MOESM1_ESM.docx]

| Comparison of CPET and hemodynamics stratified by sex and Cpc-PH or Ipc-PH | | | | | |
| --- | --- | --- | --- | --- | --- |
|  | Cpc-PH,  lower peak VO_2_/kg  (n=19) | Ipc-PH,  lower peak VO_2_/kg  (n=12) | Cpc-PH,  higher peak VO_2_/kg  (n=27) | Ipc-PH,  higher peak VO_2_/kg  (n=31) | *P*-value |
| sPAP, mmHg | 75.0 (60.0, 97.0) * | 47.5 (44.3, 61.3) | 68.0(60.0, 84.0) | 45.0(39.0, 50.0) ** | < 0.001 |
| dPAP, mmHg | 26.0(19.0, 31.0) * | 14.0(13.0, 19.8) | 23.0(20.0, 26.0) | 15.0(12.0, 18.0) ** | < 0.001 |
| mPAP, mmHg | 46.0(36.0, 50.0) * | 29.0(26.0, 33.8) | 42.0(36.0, 47.0) | 27.0(24.0, 32.0) ** | < 0.001 |
| PAWP, mmHg | 19.0(16.0, 23.0) | 18.0(16.0, 21.5) | 20.0(17.0, 23.0) | 18.0(16.0, 19.0) | 0.253 |
| PVR, Wood U | 5.3(3.5, 6.8) * | 2.1(1.7, 2.7) | 4.4(3.8, 5.3) | 2.0(1.4,2.5) ** | < 0.001 |
| DPG, mmHg | 4.0(1.0, 12.0) * | -2.5(-6.8 1.8) | 2.0(0, 7.0) | -3.0(-5.0, -1.0) ** | < 0.001 |
| TPG, mmHg | 25.0(16.0, 33.0) * | 11.0(9.0, 14.5) | 20.0(18.0, 25.0) | 10.0(7.0, 13.0) ** | < 0.001 |
| CO, L/min | 4.5(4.0, 5.4) | 5.6(5.0, 6.4) | 4.7(3.9, 5.4) | 5.4(4.1, 6.2) ^#^ | 0.027 |
| Workload, watts | 30.7 ± 21.6 ** | 31.7 ± 19.3 | 67.3 ± 33.1 | 66.6 ± 29.4 * | < 0.001 |
| Peak O_2_ pulse, ml/beat | 4.8 ± 1.5 ** | 5.8 ± 1.5 | 7.0 ± 2.4 | 7.1 ± 2.0 | < 0.001 |
| Exercise Time, s | 180.0(100.0, 220.0) ** | 155.0(97.5, 177.5) | 252.0(220.0, 290.0) | 250.0(190.0, 320.0) * | < 0.001 |
| Peak VO_2_, mL/min/kg | 8.1 ± 1.7 ** | 9.1 ± 1.1 | 13.8 ± 2.0 | 14.6 ± 2.4 * | < 0.001 |
| Lowest VE/VCO_2_ | 46.4(43.2, 56.0) ^#&^ | 40.6(35.7, 42.3) | 40.2(33.0, 44.3) | 35.2(31.4,37.6) ^#&^ | <0.001 |
| VE/VCO_2_ Slope | 50.3(35.2, 61.9) ^#&^ | 33.1(27.9, 35.5) | 32.2(28.9, 40.0) | 31.1(27.1, 35.7) | 0.002 |
| Peak VE/VCO_2_ | 49.1(43.7, 56.8) *^#^ | 40.4(35.9, 42.5) | 41.0(34.6, 49.1) | 35.8(32.7, 38.6) ^#&^ | < 0.001 |
| Peak P_ET_ CO_2_, mmHg | 27.0 ± 5.8 *^#^ | 35.2 ± 3.8 | 33.8 ± 7.7 | 35.9 ± 4.4 | < 0.001 |
| Peak VO_2_/VE, mL/L | 20.2 ± 5.0 ^#&^ | 26.2 ± 3.7 | 24.9 ± 6.2 | 27.1 ± 4.7 | < 0.001 |
| OUEP, mL/L | 23.9 ± 4.2 ^#&^ | 28.4 ± 2.8 | 27.7 ± 5.5 | 30.5 ± 3.9 ^#^ | < 0.001 |
| OUES | 0.8 ± 0.3 ** | 1.0 ± 0.3 | 1.4 ± 0.5 | 1.4 ± 0.4 ^&^ | < 0.001 |
| Abbreviations: sPAP, systolic pulmonary artery pressure; dPAP, diastolic pulmonary artery pressure; mPAP, mean pulmonary artery pressure; PAWP, pulmonary artery wedge pressure; PVR, pulmonary vascular resistance; DPG, diastolic pulmonary pressure gradient; TPG, transpulmonary gradient; CO, cardiac output; VO_2_, oxygen uptake; VE/VCO_2_, minute ventilation/carbon dioxide output; P_ET_ CO_2_, end-tidal partial pressure of CO_2_; VO_2_/VE, oxygen uptake/minute ventilation; OUEP, oxygen uptake efficiency plateau; OUES, oxygen uptake efficiency slope.  **P*<0.001 versus Ipc-PH, lower peak VO_2_/kg; ***P*<0.001 versus Cpc-PH, higher peak VO_2_/kg  ^&^*P*<0.05 versus Ipc-PH, lower peak VO_2_/kg; ^#^*P*<0.05 versus Cpc-PH, higher peak VO_2_/kg | | | | | |
